# Fatal drug poisonings in a Swedish general population

**DOI:** 10.1186/1472-6904-9-7

**Published:** 2009-04-27

**Authors:** Anna K Jönsson, Olav Spigset, Micaela Tjäderborn, Henrik Druid, Staffan Hägg

**Affiliations:** 1 Nordic School of Public Health, Gothenburg, Sweden; 2 Division of Clinical Pharmacology, Linköping University, Linköping, Sweden; 3 Division of Clinical Pharmacology, St. Olav University Hospital, Trondheim, Norway; 4 Department of Laboratory Medicine, Children's and Women's Health, Norwegian University of Science and Technology, Trondheim, Norway; 5 Department of Forensic Medicine, Karolinska Institute, Stockholm, Sweden

## Abstract

**Background:**

Pharmaceutical drug poisonings have previously been reported using single sources of information, either hospital data or forensic data, which might not reveal the true incidence. We therefore aimed to estimate the incidence of suspected fatal drug poisonings, defined as poisonings by pharmaceutical agents, by using all relevant case records from various sources in a Swedish population.

**Methods:**

Every seventh randomly selected deceased in three counties in southeastern Sweden during a one-year period was identified in the Cause of Death Register. Relevant case records (death certificates, files from hospitals and/or primary care centres and medico-legal files) were reviewed for all study subjects.

**Results:**

Of 1574 deceased study subjects, 12 cases were classified as pharmaceutical drug poisonings according to the death certificates and 10 according to the medico-legal files. When reviewing all available data sources, 9 subjects (0.57%; 95% confidence interval: 0.20–0.94%) were classified as drug poisonings, corresponding to an incidence of 6.5 (95% confidence interval: 2.3–10.7) per 100 000 person-years in the general population. The drug groups most often implicated were benzodiazepines (33%), antihistamines (33%) and analgesics (22%).

**Conclusion:**

Fatal drug poisonings is a relatively common cause of death in Sweden. By using multiple sources of information when investigating the proportion of fatal poisonings in a population, more accurate estimates may be obtained.

## Background

Drug poisonings, by pharmaceutical agents, have been reported to comprise approximately 1% of emergency department visits [1-3] and up to 0.4% of these result in a fatal outcome [1,3]. Among the deceased subjected to a forensic autopsy, 0.2% of the deaths have been reported to be caused by pharmaceutical drug poisonings [4]. The incidence of fatal pharmaceutical drug poisonings in a general population has previously been estimated to vary between 0.14 and 5.3 per 100 000 inhabitants based on data from single sources of information, such as hospital data [5,6] or forensic data [4,7-9]. The incidence of fatal drug poisonings in a general population has previously been estimated based on the official cause of death statistics [10-12]. However, in these compilations, pharmaceutical drug poisonings were not analysed separately. Since it can be expected that not all cases of fatal drug poisonings will be identified by using one single data source, these studies might not be able to reveal the true incidence. We therefore aimed to estimate the incidence of suspected fatal drug poisonings, defined as poisonings by pharmaceutical agents, by using all relevant case records from various sources in a Swedish population. Another purpose of the study was to estimate the number of cases reported by the different types of case records included.

## Methods

This was a retrospective study conducted in three counties in southeastern Sweden – Östergötland, Jönköping and Kalmar counties – comprising a mean population of 974 884 inhabitants during the study period, 1 January 2001 to 31 December 2001. Approval of this study was obtained from the Swedish National Board of Health and Welfare. All 11 015 fatalities in the area were identified according to their annual report; Causes of Death 2001 [13]. One out of every seventh deceased in the population was selected at random and the death certificates were retrieved. Since all Swedish residents receive a unique personal identification number, it is possible to link the information from the death certificates to other case records. The data extraction procedure has previously been described in detail elsewhere [14]. In short, a thorough drug history for all 1574 subjects focusing particularly on the 14 days preceding death was obtained from relevant case records (medical charts from hospitals and/or primary care centres, death certificates and medico-legal files). During this first examination, performed by four health care professionals, relevant information was extracted to a standardised form. Further evaluation of possible fatal poisonings and their causality was performed by two pharmacists, two clinical pharmacologists and a forensic pathologist in accordance with WHO criteria [15]. A case was categorised as drug poisoning if there was a relationship between death and drug toxicity which was assessed as at least *possible *(known toxicity of the drug(s) suspected and reasonable time between intake and death but other contributory causes of death may exist). For an event to be classified as a drug poisoning, consensus had to be reached between all assessors.

Drug poisonings were defined as poisonings by pharmaceutical agents, and were categorised as being intentional, unintentional or with unknown intent. More than one drug could have been involved in the poisoning of a single case. In fatalities where a forensic autopsy investigation had been performed (in unnatural deaths and suspected unnatural deaths as a part of the post-mortem investigation), femoral blood, urine and vitreous samples were collected and screened for drugs and ethanol. In subjects in whom drugs were detected, compilations of fatal and non-fatal concentrations of drugs in post-mortem femoral blood was used to support the causality assessment [16,17] in addition to the perusal of the history and the description of the circumstances surrounding death in each case. To control for possible misclassification from the first assessment, we reviewed every tenth of the fatalities not categorised as drug poisonings.

Statistical analyses were performed using the statistical program package STATA (STATA/IC 10.0 for Windows; Statacorp, TX, US). The analyses included Fischer exact tests for dichotomous variables and Mann-Whitney tests for continuous variables. *P*-values < 0.05 were considered statistically significant.

## Results

The death certificates were retrieved for all 1574 subjects and in 99% of the total study population (1553 subjects), information about drug prescriptions during the last year before death could be retrieved. Medical case records were available for 1503 subjects (95%) and forensic autopsies were carried out in 83 subjects (5.3%), and medico-legal files were available in all of these subjects. Based on the information from the death certificates, 12 fatalities of the 1574 deceased study subjects were classified as pharmaceutical drug poisonings. Based on the information from the medico-legal files, 10 fatalities were classified as pharmaceutical drug poisonings. When reviewing all available data sources, 9 subjects (0.57%; 95% confidence interval: 0.20–0.94%) were classified as drug poisonings, corresponding to an incidence of 6.5 (95% confidence interval: 2.3–10.7) per 100 000 person-years in the general population. The characteristics of the study population are shown in Table 1. The drug groups most often implicated were benzodiazepines (33%), antihistamines (33%) and analgesics (22%) (Table 2). Of the 9 poisonings, 4 could be classified as intentional, 3 as unintentional, and 2 as having unknown intent. When reviewing every tenth of the 1565 fatalities not classified as drug poisonings, no additional fatal drug poisonings were identified.

**Table 1:** Characteristics of 1574 deceased subjects in a random sample of the Swedish population

Variable	Subjects with pharmaceutical drug poisonings (n = 9)	Remaining subjects (n = 1565)	P value
Number of males (%)	5 (56%)	806 (51.5%)	1.0
Age, years, median (range)			
Males	53 (26–85)	79 (0–101)	0.08
Females	49 (27–85)	84 (0–104)	<0.05
Forensic autopsy performed, n (%)	7 (78%)	76 (4.9%)	<0.01
Clinical autopsy performed, n (%)	0 (0%)	115 (7.3%)	1.0
Fatalities in hospital, n (%)	3 (33%)	636 (40.6%)	0.8

**Table 2:** Details of the 9 fatalities classified as pharmaceutical drug poisonings

Case number	Sex	Age	Type of drug poisoning	Suspected drugs	Postmortem femoral blood concentration (μg/g)
1	Female	81	Intentional	Nitrazepam	NA
2	Female	34	Intentional	Levomepromazine	3.6
3	Female	27	Intentional	Morphine	0.18
4	Male	26	Intentional	Nitrazepam	0.7^a^
				+ carbamazepine	17
				+ propoxyphene	1.2
				+ promethazine	0.1
5	Male^b^	85	Unintentional	Digoxin	NA^c^
6	Female	64	Unintentional	Amitriptyline	3
7	Male^d^	53	Unintentional	Alimemazine	3.9
8	Male	80	Unknown intent	Diazepam	0.4
9	Male^e^	53	Unknown intent	Alimemazine	0.7

*Abbreviation*: NA = not available

^a ^Analysed as the metabolite 7-amino-nitrazepam

^b ^This case was also suspected to have an adverse drug reaction contributing to the fatal outcome, a warfarin-related intravesical haemorrhage [14].

^c ^The plasma concentration of digoxin was 6.9 mmol/l at admission to hospital.

^d ^1.5 ‰ of ethanol was detected in postmortem urine.

^e ^4.4 ‰ of ethanol was detected in postmortem femoral blood.

## Discussion

In the present study, which identified subjects by means of a review of death certificates as well as medical charts from hospital and/or ambulatory care and medico-legal files, 0.57% of the deaths were considered to be caused by drug poisoning, corresponding to an incidence of 6.5 fatal poisonings per 100 000 person-years. This proportion is in accordance with the data in the Swedish forensic pathology and toxicology databases, where about 550 fatal poisonings are registered annually (unpublished observations). Given that Sweden has 9 million inhabitants, this figure corresponds to an incidence of 6.1 per 100 000 inhabitants. Published reports regarding the incidence of fatal poisonings by a pharmaceutical agent in a general population from other countries have been based on either hospital data or medico-legal data. The incidence of fatal drug poisonings has been estimated based on hospital data to 0.35 per 100 000 person-years in Iceland [5] and 0.54 per 100 000 person-years in Finland [6]. From forensic data, the incidence of fatal drug poisonings has been estimated to vary between 1.9 and 5.3 per 100 000 person-years in the US [7], 2.5 per 100 000 person-years in Canada [8], 0.1 per 100 000 person-years in Greece [4] and 2.0 per 100 000 person-years in New Zealand [9]. The variations observed may easily be explained by regional differences, and the different kinds of data sources used. The source of information used when studying fatal drug poisonings may not only influence the proportion of cases observed in a population but also the observed drugs suspected to have caused fatal poisonings [18]. Hence, it is important to combine the information from different data sources when investigating the proportion of fatal poisonings in a population.

In Sweden, the information on the death certificates for all fatalities are registered in the Cause of Death Register; in our study, all the nine fatal drug poisonings as well as three additional cases were reported as drug poisonings on the death certificates. Therefore, the information on the death certificates may be a valuable means to identify fatal poisonings, although this approach is clearly dependent on the quality of the examinations performed to ascertain the cause(s) of death and the quality of the ICD (International Classification of Diseases) coding on the death certificates [19,20]. Moreover, to identify the substances responsible for the poisonings, additional information, e.g. from medical charts, is needed since the ICD codes on the death certificates do not always specify the individual substance(s) taken. Another pitfall when using information on the death certificates only is that several drugs may be mentioned without describing their relative contribution to the poisoning.

Data registered based on forensic autopsies may be another valuable source, since in Sweden most subjects with suspected fatal poisonings are subjected to a forensic autopsy, 78% of the cases in this study. In addition, a toxicological screening of alcohol, pharmaceutical drugs and illicit drugs are performed in most of these cases. However, post-mortem degradation [21] and redistribution [22,23] may significantly alter blood drug levels after death. The concentrations can vary between sampling sites, and may change over time, particularly at high ambient temperatures. To reduce these errors that affect some drugs more than others, a standardised procedure for sampling, handling and analysis of toxicological samples was introduced at all forensic medicine departments in Sweden in 1990.

One limitation of the present study is its retrospective design. Despite scrutinising the medico-legal files, medical charts, police reports and additional sources of information, it is impossible to obtain all relevant information in each case; hence the number of drug poisonings might have been underestimated. Moreover, the number of fatal poisonings observed in the study is limited. On the other hand, this study has several strengths compared with previous studies. The population based approach includes a random selection of all deceased subjects in the study area. In 99% of the cases, it was possible to follow the general health and drug histories over a relevant period of time prior to death by consulting various sources. Furthermore, the application of a multidisciplinary approach during evaluation of the suspected drug poisonings reduces the risk of misclassification. Cases with a more likely alternative explanation for the death, with uncertainties or inconsistencies related to the information found were excluded as suspected drug poisonings. Using this conservative approach, consensus was achieved for all cases included.

Benzodiazepines, antihistamines and opioid analgesics were most commonly associated with fatal poisonings in this study but the numbers in each group were small. The substances detected may reflect their inherent toxicity, but also their sales and overall use [24,25], which vary between geographical areas [26]. The present study verifies that drug groups commonly involved in fatal drug poisonings include analgesics, antidepressants and sedatives in Europe. The same pattern has been seen in North America, but is not necessarily present in other areas of the world [1,7].

## Conclusion

In conclusion, fatal drug poisonings are a relatively common cause of death in Sweden. By using multiple sources of information when investigating the proportion of fatal poisonings in a population, more accurate estimates may be obtained.

## Competing interests

The authors declare that they have no competing interests.

## Authors' contributions

AKJ participated in the design of the study, carried out data extraction, took part in the assessment of cases and drafted the manuscript. OS and HD took part in the assessment of cases and revised the manuscript. MT revised the manuscript. SH participated in the design of the study, took part in the assessment of cases and revised the manuscript. All authors have also made substantive intellectual contribution to the study and have approved the final manuscript.

## Pre-publication history

The pre-publication history for this paper can be accessed here:


